# Association of the polymorphism Exon 1 (A/O) region of the mannose-binding lectin gene and periportal fibrosis regression in schistosomiasis after specific treatment

**DOI:** 10.1590/0037-8682-0145-2020

**Published:** 2020-12-21

**Authors:** Saulo Gomes de Oliveira, Ilana Brito Ferraz de Souza, Taynan da Silva Constantino, Paula Carolina Valença Silva, Elker Lene Santos de Lima, Maria Tereza Cartaxo Muniz, Ana Lúcia Coutinho Domingues

**Affiliations:** 1 Universidade Federal de Pernambuco, Centro Acadêmico de Vitória, Vitória de Santo Antão, PE, Brasil.; 2 Universidade de Pernambuco, Centro de Oncohematologia Pediátrica, Recife, PE, Brasil.; 3 Universidade de Pernambuco, Instituto de Ciências Biológicas, Recife, PE, Brasil.; 4 Universidade Federal de Pernambuco, Departamento de Medicina Clínica, Recife, PE, Brasil.

**Keywords:** Schistosomiasis, Periportal ﬁbrosis, Mannose-binding lectin

## Abstract

**INTRODUCTION:**

We evaluated the association between genetic polymorphisms in exon 1 (A/O alleles) and promoter regions at positions -550 (H/L variant, rs11003125) and -221 (X/Y variant, rs7096206) *MBL2* and periportal fibrosis regression.

**METHODS:**

This was a retrospective cohort study involving 114 Brazilians infected with *Schistosoma mansoni*, who were subjected to follow-up for three years after specific treatment for schistosomiasis to estimate the probability of periportal fibrosis regression.

**RESULTS:**

A risk association was observed between polymorphism at the exon 1 *MBL2* and periportal fibrosis regression.

**CONCLUSIONS:**

This study suggests that the polymorphism of exon 1 *MBL2* may potentially be used to predict periportal fibrosis regression in this population.

Periportal fibrosis (PPF) is the major pathological consequence of schistosomiasis. PPF is represented by an inflammatory and fibrotic response that occurs due to the presence of *Schistosoma mansoni* (SM) eggs in the liver, which can cause portal hypertension and, consequently, lead to the rupture of esophageal varices^1^.

Some factors, such as consumption of alcohol, exposure to SM*-*contaminated areas (includes one-time exposure), specific treatment for SM, age, sex, and nutritional status of the individual may influence the progression of PPF. Additionally, the host immune response is considered an important factor in PPF pathogenesis^2^.

In the fibrogenesis process, mannose-binding lectin (MBL) is related to the development of PPF^3^. This protein plays an important role in activation of the complement system by binding the MBL/serine protease associated with MBL (MBL/MASP) and opsonization. MBL levels are higher in individuals with advanced periportal ﬁbrosis^3^ ^,^ ^4^.

Some polymorphisms identified in the promoter region of *MBL2* -550 (H/L variant guanine to cytosine (G-C) nucleotide substitutions, rs11003125) and -221 (X/Y *X/Y* variant, C to G nucleotide substitution, rs7096206) and in the 5-untranslated region of exon 1 at position +4 (A/O variant, C to T nucleotide substitution (c.4T/C) or rs7095891) are responsible for functional alterations and influence the circulating concentration of MBL^5^.

In the exon 1 region of *MBL2*, three polymorphisms are present in codons 52 (rs5030737), 54 (rs1800450), and 57 (rs1800451), which lead to the formation of three allelic variants, namely D, B, and C. These variant alleles are grouped into the ‘‘O’’ allele, and the normal allele is represented by ‘‘A’’^6^ ^,^ ^7^.

Additionally, in the promoter region of the *MBL2* gene, mutations arise in the -550 region, which lead to the formation of H/L alleles, where the nucleotide alteration from G to C occurs, and at position -221, X/Y alleles, wherein a similar nucleotide alteration occurs^8^. The combination of alleles from these different regions of the *MBL2* is called a haplotype. The union of alleles of the promoter region and exon 1 generates common haplotypes (HYPA, LYQA, LYPA, LXPA, LYPB, LQC, and HYPD)^7^.

Therefore, the objective of this study was to investigate the association between clinical factors (sex, upper gastrointestinal bleeding (UGB), specific treatment, and contact with focus area) and polymorphisms of the exon 1 region as well as the promoter regions (-550 and -221) of the *MBL* with PPF regression after specific treatment in patients with SM in populations of endemic areas in the state of Pernambuco, northeastern Brazil.

This was a retrospective cohort study conducted from August 2016 to February 2019 to verify the association between *MBL2* gene polymorphisms and PPF regression.

A total of 114 patients infected with SM, who were inhabitants of an endemic area for schistosomiasis in the state of Pernambuco, northeastern Brazil, were enrolled and their cases were analyzed for three years retrospectively, before and after specific treatment for SM, to estimate the likelihood of PPF regression. A decrease in one pattern of PPF was maintained in subsequent ultrasound for three years.

 All patients were divided into the following two groups: Group 1, exposed patients with genotypes (OO/AO exon 1; -550 HL, HH and -221 XY, XX); Group 2, not exposed patients with genotypes (AA exon 1; -550 LL and -221 YY). All individuals were older than 18 years and were assisted at the Outpatient Gastroenterology Clinic of the Hospital das Clinicas, Federal University of Pernambuco (HC-UFPE), Brazil.

This study included subjects diagnosed with the hepatosplenic form of the disease with advanced PPF (pattern E or F) or with the hepatointestinal form of the disease with mild or moderate PPF (pattern C or D). The pattern of PPF was evaluated by abdominal ultrasound, using the Niamey^9^ protocol before and three years after treatment for schistosomiasis. This protocol classifies PPF cases based on patterns from A (normal) to F (very advanced)^9^.

Individuals with other associated liver diseases (hepatitis B, hepatitis C, alcoholic disease, steatosis) described in medical records and those with other clinical forms of schistosomiasis (pulmonary vascular disorders, pseudoneoplastic forms, schistosomal nephropathy, and schistosomal myeloradiculopathy) were excluded from this study.

The outcome of interest was the pattern of PPF regression identified by ultrasonography of the upper abdomen. The main exposures evaluated were polymorphisms in the exon 1 region and promoter regions -550 and -221 of the *MBL2* gene, which, in the context of the hypothesis of this study, were investigated for associations with PPF regression. The pattern regression of PPF was considered the dependent variable.

Information on these genotypes and haplotypes was obtained in 2012 and 2013^3^. Single-nucleotide polymorphisms of the *MBL2* gene were detected by real-time polymerase chain reaction (RT-PCR) and haplotypes were determined according to the methods reported by Garred et al.^10^.

Other explanatory variables were gender, age, diagnosis of serious UGB, and contact with contaminated water by SM during the study period. Data were collected using a precoded structured questionnaire that was used for individuals by a single investigator.

Follow-up was initiated from the period the specific treatment was first recorded and ended three years later. Crude relative risk (RR) and 95% confidence interval (95% CI) were used through univariate analysis (Chi-squared Yates corrected). The significance level was set at 0.05. Epi Info version 3.5.5 (CDC, Atlanta, GA, USA) was used for all statistical analyses.

The study was conducted according to the Declaration of Helsinki and approved by the Ethics and Research Committee of the Health Sciences Center of the Federal University of Pernambuco under protocol 113.199 and CAAE 03161512.6.0000.5208.

In this study, 139 patients were initially selected for convenience, of which 24 were excluded because they were not subjected to pre- and post-specific treatment ultrasound follow-up, and one was excluded due to associated pulmonary hypertension. Eight patients were excluded as there was no information available regarding the fibrosis pattern. Ultimately, 106 patients were enrolled in this study (Figure 1). As a few DNA samples obtained from six patients could not be amplified, they were not specifically analyzed for RT-PCR in the promoter regions (-550 and -221) and structural exon 1 of the *MBL2* gene.

**FIGURE 1:**
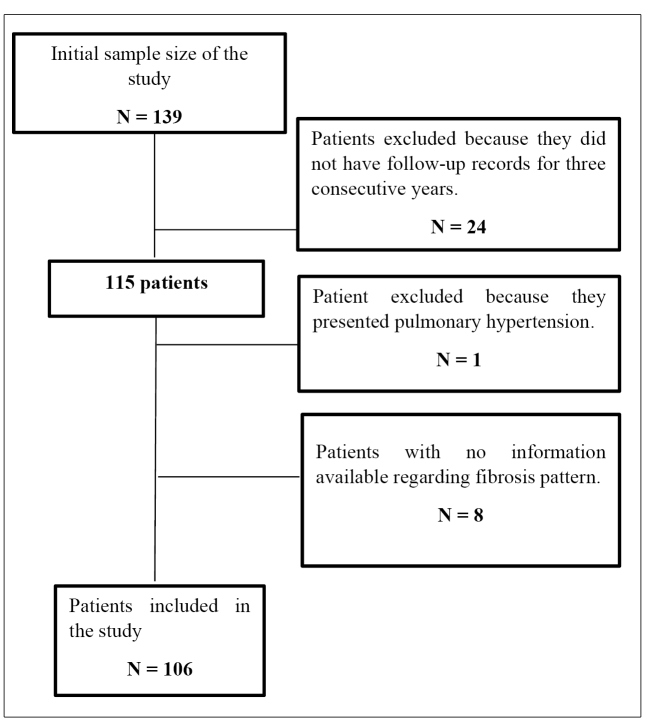
Patient eligibility fluxogram.

There was no difference in gender predominance, and the mean age was 57 years (SD: 11 years). In 64 (60%) patients, PPF was stable, in 21 (20%) decreased and in 21 (20%) increased. There was no association between sociodemographic and clinical variables and the PPF pattern of regression (Table 1).

A risk association was observed among individuals with *MBL2* OO/AO genotypes (low and intermediate MBL expression, respectively; RR = 2.450; CI = 1.200-4.990, p-value = 0.015) and image pattern of PPF regression when compared to individuals with the AA genotype (high MBL expression) (Table 1). There was no association between promoter regions -550 and -221 *MBL2* (RR = 1.035; CI = 0.853-1.257, p-value = 0.468 and RR = 0.893, CI = 0.741-1.077, p = 0.230, respectively) and MBL haplotypes and the image pattern of PPF regression (Table 1).

**TABLE 1:** Association between sociodemographic and clinical variables, the *MBL2* gene polymorphisms in the promoter (-550, -221) and structural regions, *MBL* haplotypes, and the regression of the periportal fibrosis pattern in patients with schistosomiasis in the state of Pernambuco, Brazil.

Regression of the periportal fibrosis pattern							
	Yes (n = 21)		No (n = 85)				
Characteristics	n	%	n	%	RR	CI 95%	p-value
**Sex**							
Female	12	57	44	52	1	(reference)	
Male	9	43	41	48	0.958	[0.793 - 1.157]	0.421
Total	21	100	85	100			
**HDA**							
No	15	71	43	51	1	(reference)	
Yes	6	29	42	49	2.069	[0.870 - 4.919]	0.070
Total	21	100	85	100			
**Treatment for HDA**							
Not treated	14	67	39	46	1	(reference)	
Treated	7	33	46	54	0.847	[0.699- 1.027]	0.071
Total	21	100	85	100			
**Current contact with focus area**							
No	20	95	78	92	1	(reference)	
Yes	1	5	7	8	0.909	[0.687 - 1.204]	0.468
Total	21	100	85	100			
**Contact with focus area after treatment**							
No	17	81	73	86	1	(reference)	
Yes	4	19	12	14	1.081	[0.801 - 1.459]	0.411
Total	21	100	85	100			
**Structural region (Exon 1) *MBL***							
AA	11	52	62	73	1	(reference)	
AO/OO	10	48	23	27	2.450	[1.200 - 4.990]	0.015
Total	21	100	85	100			
**Promoter region -221 *MBL*** ^a^							
YY	16	84	59	73	1	(reference)	
XX/XY	3	16	22	27	0.893	[0.741 - 1.077]	0.230
Total	19	100	81	100			
**Promoter region -550 *MBL*** ^a^							
LL	6	32	29	36	1	(reference)	
HL/HH	13	68	52	64	1.035	[0.853 - 1.257]	0.468
Total	19	100	81	100			
***MBL* haplotypes** ^a^							
(LYO/LXO;LYO/LYO;HYA/LXO)							
High expression	8	42	42	52	1	(reference)	
(HYA/LYA)							
Intermediate expression	9	47	34	42	1.062	[0.873 - 1.291]	0.365
Low expression	2	11	5	6	1.176	[0.724 - 1.907]	0.386
(LYA/LYO;HYA/LYO;HYA/LXA)							
Total	19	100	81	100			

^a^ One hundred patients were evaluated for polymorphism in the promoter regions (-550 and -221) in relation to *MBL* haplotypes, as six patients who did not present genotyping by DNA condition were excluded. **HDA:** upper digestive hemorrhage; **EDA:** endoscopic treatment; **MBL:** mannose-binding lectin; **N:** number of patients; **RR:** relative risk; **CI:** confidence interval; p-value.

Although previous studies have established that several environmental factors (age, exposure frequency, parasite load, specific treatment, and level of education) influence the natural history of PPF^11^, no evidence has been made available, to our knowledge, regarding the statistical association between the classic factors and PPF regression.

Similarly, Silva et al.^4^ evaluated 79 individuals infected with SM who presented different PPF patterns in the state of Pernambuco. They found no evidence of an association between classic factors and the severity of PPF. This suggests that there is a need for further cohort studies to assess other risk factors not addressed in this study to further explain the environmental risk factors in this population.

Some polymorphisms identified in promoter regions and in the exon 1 region of the *MBL* gene cause functional alterations and influence the serum concentration of this protein^7^.

In this study, evidence of risk association was found between the genotypes (AO/OO) exon 1 *MBL* and the PPF pattern regression in this population, which is in agreement with reports that OO homozygotes, AO heterozygotes, and AA homozygotes exhibit low MBL concentrations, intermediate concentrations, and elevated serum MBL concentrations, respectively^7^.

Similar results were reported by Constantino et al.,^3^ who analyzed 229 individuals infected with SM in Pernambuco, northeastern Brazil, and found that MBL serum concentrations were significantly higher in individuals with advanced PPF. They also identified a protective association between the A/O genotype of exon 1 *MBL* and elevated serum concentrations of MBL. They suggested that elevated MBL levels might contribute to liver damage in schistosomiasis and might be a predictive factor for PPF severity.

Similarly, Silva et al.^4^ evaluated 79 patients infected with SM in Pernambuco, northeastern Brazil, and found that higher serum concentrations of MBL were signiﬁcantly associated with advanced PPF.

Further, Pedroso et al.^12^ analyzed the impact of polymorphisms on the promoter and exon 1 MBL regions in 102 Euro-Brazilians with chronic hepatitis C and another group of seronegative controls composed of 102 individuals in Curitiba, southern Brazil. They found that *MBL* A/O genotypes were more frequent in patients with chronic hepatitis C compared to those in healthy control patients, and that low MBL levels might reduce the risk of chronic hepatitis C.

In contrast, Erdemir et al.^13^ found controversial results when analyzing the impact of exon 1 *MBL* polymorphisms on chronic hepatitis B (CHB) in 67 children with CHB and 99 healthy controls in Turkey. They found that genotype (OO) was substantially more frequent in children with CHB and that genotype (AO) was found predominantly in healthy control patients. They concluded that the homozygous genotype (OO) was associated with the development of chronic infection and the severity of liver disease.

In this study, there was no evidence of an association between *MBL2* polymorphism promoters and the regression of PPF in this population. Considering the limitation of sample size in this study as well as possible ethnic variations in the population, further studies to explore the association between these polymorphisms and the regression of PPF are recommended.

A previous study reported that there was no association between genetic polymorphisms in exon 1 and promoter regions -221 and -550 of the *MBL* gene, with the PPF pattern. There was also no association between haplotypes of *MBL* and PPF severity. These findings might have been influenced by ethnic variations^3^.

Vallinoto et al.^14^ also investigated the impact of *MBL* gene polymorphisms on hepatitis C virus infections and compared 73 hepatitis C virus-infected patients and 92 seronegative controls in the Brazilian Amazon region. They found that there was no association between polymorphisms and disease progression. The authors considered the hypothesis that ethnic variations could have influenced these results.

The results reported in this study do not exclude the possibility that the *MBL* gene in other regions may influence PPF regression. Therefore, future studies with larger samples are warranted to better analyze these polymorphisms for improved assessment to ascertain an association between polymorphisms in the promoter region variants -550 and -221 *MBL2* and PPF intensity, which may influence PPF regression after specific treatment for schistosomiasis.

Overall, we identified a risk association between the polymorphism at exon 1 *MBL2* and PPF regression. Our results suggest that the polymorphism of exon 1 *MBL2* may potentially be used to predict PPF regression in the Brazilian population.
